# Single-cell RNA sequencing reveals critical modulators of extracellular matrix of penile cavernous cells in erectile dysfunction

**DOI:** 10.1038/s41598-024-56428-0

**Published:** 2024-03-11

**Authors:** Chao Luo, Yaqian Peng, Jiang Gu, Tao Li, Qiang Wang, Xiaolan Qi, Anyang Wei

**Affiliations:** 1 Department of Urology, Affiliated Hospital of Guizhou Medical University, Guiyang, 550004 Guizhou China; 2 Key Laboratory of Endemic and Ethnic Diseases, Ministry of Education and Key Laboratory of Medical Molecular Biology of Guizhou Province, Guizhou Medical University, No. 9 Beijing Road, Yunyan District, Guiyang City, 550004 Guizhou Province China; 3 Department of Urology, Nanfang Hospital, Southern Medical University, No.1838 Guangzhou Avenue North, Guangzhou City, Guangdong Province China

**Keywords:** Single-cell sequencing, Erectile dysfunction, Extracellular matrix, Microenvironment, Modulator, Computational biology and bioinformatics, Genetics, Molecular biology, Biomarkers, Diseases, Health care, Urology

## Abstract

Erectile dysfunction (ED) is a common and difficult to treat disease, and has a high incidence rate worldwide. As a marker of vascular disease, ED usually occurs in cardiovascular disease, 2–5 years prior to cardiovascular disease events. The extracellular matrix (ECM) network plays a crucial role in maintaining cardiac homeostasis, not only by providing structural support, but also by promoting force transmission, and by transducing key signals to intracardiac cells. However, the relationship between ECM and ED remains unclear. To help fill this gap, we profiled single-cell RNA-seq (scRNA-seq) to obtain transcriptome maps of 82,554 cavernous single cells from ED and non-ED samples. Cellular composition of cavernous tissues was explored by uniform manifold approximation and projection. Pseudo-time cell trajectory combined with gene enrichment analysis were performed to unveil the molecular pathways of cell fate determination. The relationship between cavernous cells and the ECM, and the changes in related genes were elucidated. The CellChat identified ligand-receptor pairs (e.g., PTN-SDC2, PTN-NCL, and MDK-SDC2) among the major cell types in the cavernous tissue microenvironment. Differential analysis revealed that the cell type-specific transcriptomic changes in ED are related to ECM and extracellular structure organization, external encapsulating structure organization, and regulation of vasculature development. Trajectory analysis predicted the underlying target genes to modulate ECM (e.g., COL3A1, MDK, MMP2, and POSTN). Together, this study highlights potential cell–cell interactions and the main regulatory factors of ECM, and reveals that genes may represent potential marker features of ED progression.

## Introduction

Sexual desire mediates the interplay between physiological and psychological factors. This explains why numerous patients who recover from erectile dysfunction (ED) continue to require therapy. In fact, regular and chronic ED increases with age, from approximately 35% of men over 60 years old to approximately 50% of men over 70 years of age^1^. Pathological hallmarks of ED include decreased cyclic guanosine monophosphate (cGMP) concentrations, hypoxia, endothelial dysfunction, smooth muscle cell relaxation disorder, and disturbances in nitric oxide (NO) metabolism. NO-enhancing drugs, such as phosphodiesterase 5 inhibitor (PDE-5i), can delay the decomposition of NO and are widely used in clinical practice. However, they are still not completely effective^2^. Furthermore, new drug discovery is usually an inefficient process characterized by rising costs, long lead times, and high loss rates^3^. Part of the reason of inefficiencies is our limited understanding of human biology, particularly the heterogeneity of disease related mechanisms, actionable therapeutic targets, and disease responses^3^. Therefore, in-depth research on the biological characteristics of ED patients has important clinical significance.

Traditional transcriptomics research, typically focusing on mixed cell populations, lacks the resolution necessary to accurately identify specific cell types. In contrast, single-cell sequencing data enables the capture of cellular variation, providing a deeper understanding of biological heterogeneity, which is crucial for enhancing disease stratification and treatment approaches. Recent studies employing single-cell RNA sequencing (scRNA-seq) have investigated transcriptomic alterations in ED penile tissues^4,5^, uncovering molecular changes at the individual cell level. Although these findings are significant for researchers in this domain, certain aspects remain inadequately explained. For instance, alterations in ECM signaling pathways have been observed during ED progression. Nevertheless, the regulation of these pathways in the ECM context requires more comprehensive investigation.

Studies have shown that the elasticity or stiffness of the ECM affects basic cellular processes including diffusion, growth, proliferation, migration, differentiation, and organogenesis^6^. In addition, the ECM plays an important role in angiogenesis, neuroinflammation, ischemic heart failure, and other diseases^7–9^. Penile fibrosis is a diffuse fibrosis process in the cavernous body of the penis. It is the result of various diseases related to ED, such as diabetes, atherosclerosis, iatrogenic pelvic nerve injury (after radical resection of cancer and prostate cancer), and even age-related ED^10^. Numerous cell types, including vascular endothelial cells, immune cells, and fibroblasts, are recognized for their role in fibrosis development. Specifically, fibroblasts are the principal cell type involved in penile fibrosis, contributing to ECM accumulation and inflammation. ScRNA-seq analysis has been employed to explore the heterogeneity of fibroblasts in various fibrotic conditions, such as pulmonary fibrosis, systemic sclerosis, and Dupuytren's disease^11^. Consequently, employing scRNA-seq technology to investigate the impacts of diverse cell types within cavernous tissue on the ECM could represent a promising research avenue for further elucidating the biological alterations associated with ED.

The dysfunction of endothelial cells (ECs) impacts the contractile and relaxation functions of smooth muscle cells (SMCs), a critical factor contributing to erectile dysfunction. Gaining a comprehensive understanding of intercellular communication within the corpus cavernosum of the penis is crucial for both pathological and physiological studies of erectile dysfunction. CellChat, a tool for cell–cell interaction analysis in scRNA-seq, enables the quantitative inference and analysis of intercellular communication networks from scRNA-seq data. It utilizes network analysis and pattern recognition techniques to predict the principal signaling inputs and outputs of cells and to understand how these cells and signals synergize in their functions. Employing diverse learning methods and quantitative comparisons, CellChat categorizes signaling pathways and delineates both conserved and context-specific pathways across different datasets^12^.

In this study, we profiled scRNA-seq to obtain transcriptome maps of 82,554 cavernous single cells from ED and non-ED samples. Cellular composition of cavernous tissues was explored by uniform manifold approximation and projection. Pseudo-time cell trajectory combined with gene enrichment analysis were performed to unveil the molecular pathways of cell fate determination. The relationship between cavernous cells and the ECM, and the changes in related genes were elucidated. Here, we use the CellChat that identified ligand-receptor pairs (e.g., PTN-SDC2, PTN-NCL, and MDK-SDC2) among the major cell types in the cavernous tissue microenvironment. Differential analysis revealed that the cell type-specific transcriptomic changes in ED are related to ECM, etc. Trajectory analysis predicted the underlying target genes to modulate ECM. Together, this study highlights potential cell–cell interactions and the main regulatory factors of ECM, and reveals that genes may represent potential marker features of ED progression.

## Results

### Single-cell transcriptome profiling of cavernous tissue in ED and non-ED samples

**Figure 1:**
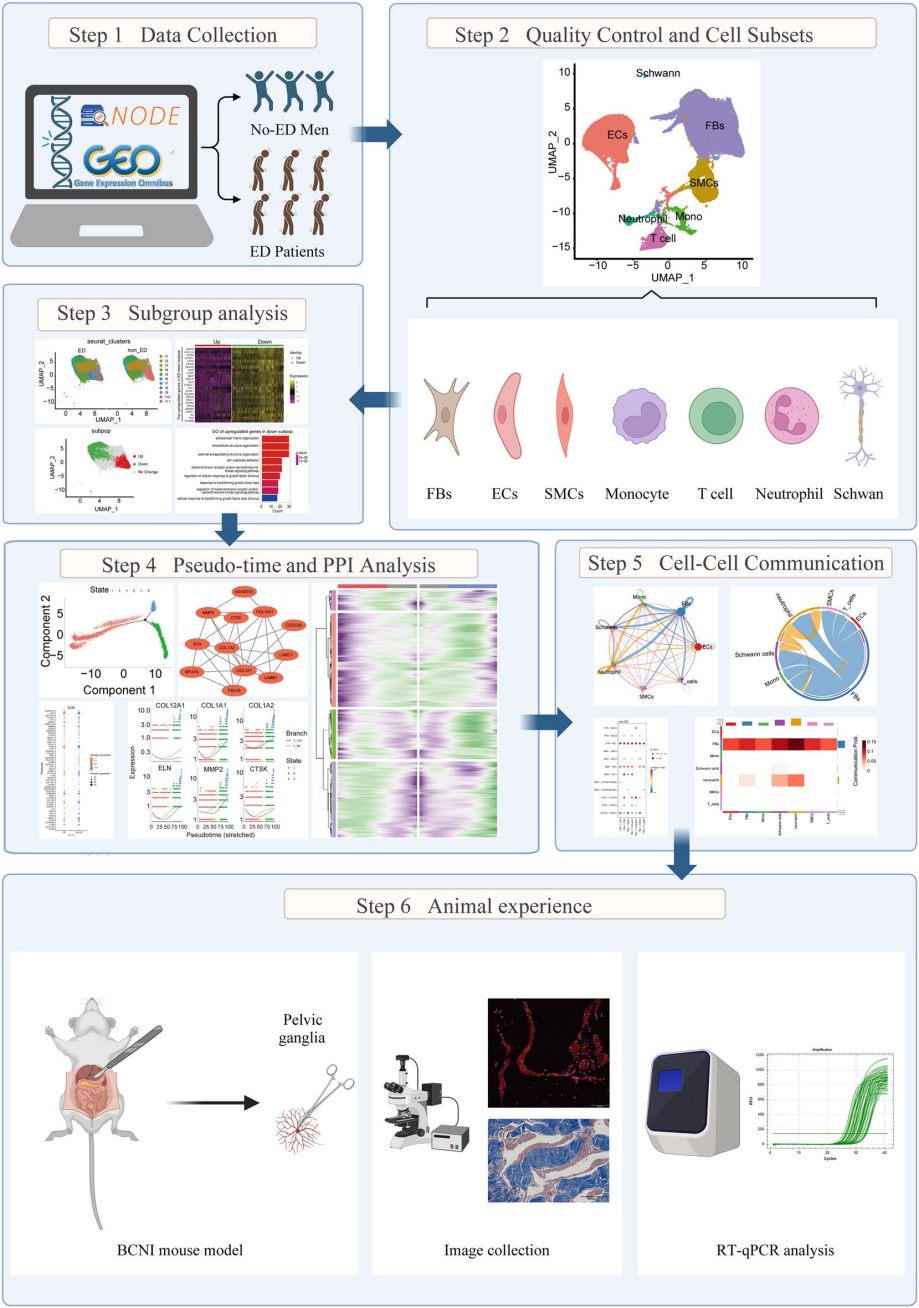
Schematic representation of the workflow used in this study.

**Figure 2:**
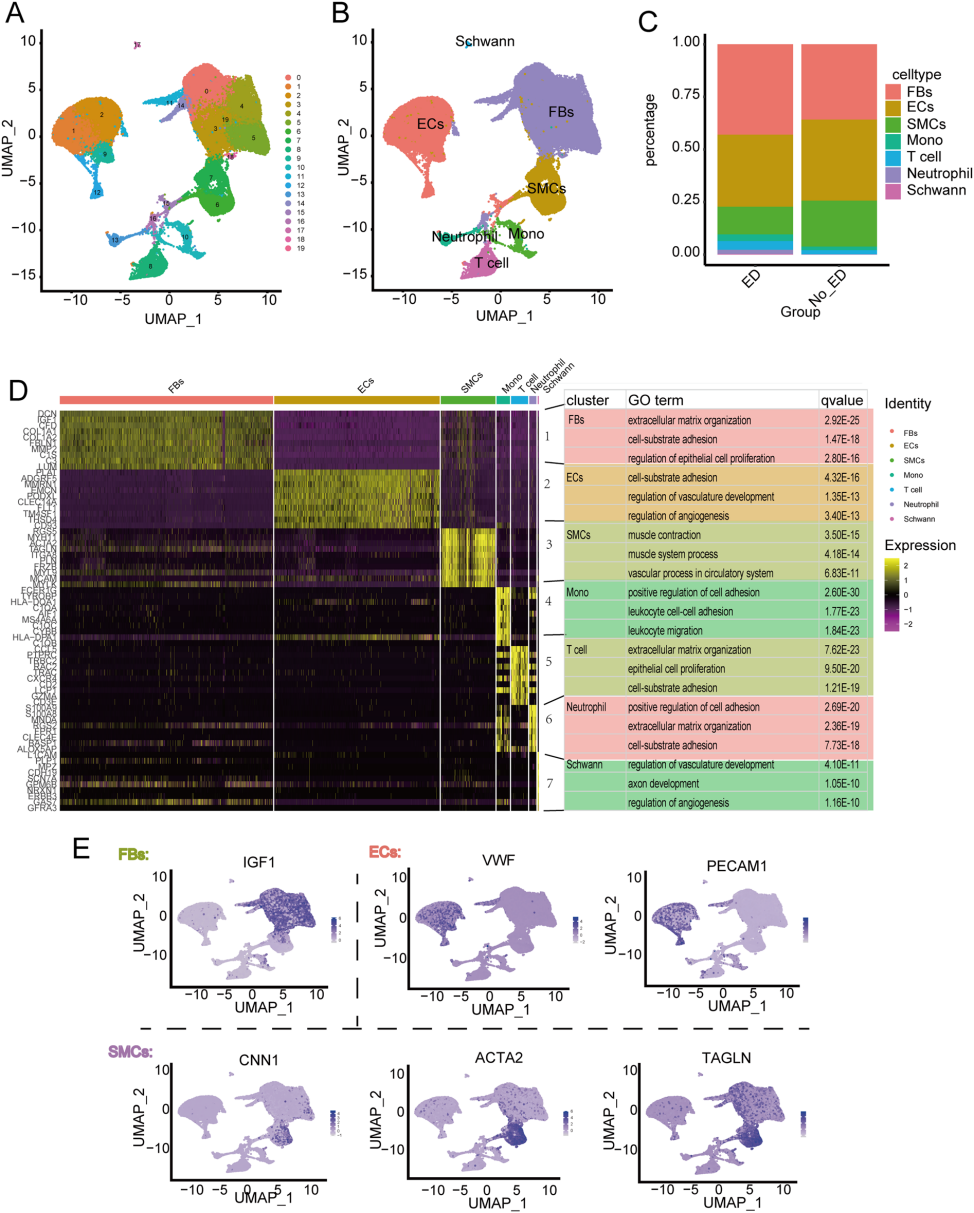
ED progression differentially affects the cell-type composition of the corpus cavernosum. (**A**) UMAP plot showing nine well-converged datasets, with six ED samples and three non-ED samples, and (**B**) depicting the nine major cell types separating from the corpus cavernosum, including ECs, Schwann cells, FBs, SMCs, ECs lymphocytes, monocytes, neutrophils, T cells and regulated T cells. (**C**) Each cell type exists in a different proportion of cells in separate data collections. (**D**) Heatmap showing the top ten most enriched genes of each cell type, and GO analysing of these genes, the red cluster denotes FBs, the yellow denotes ECs, the light green denotes Schwann cells, the green denotes monocytes, and the aquamarine denotes neutrophils. (**E**) Expression levels of representative marker genes in different in FBs, ECs and SMCs.

As illustrated in the flow chart in the Fig. 1, it is a schematic diagram of the workflow used in this study. To evaluate cellular diversity in the ED samples, we generated single-cell RNA sequence profiles from six ED samples and three non-ED samples based on two combined datasets (Fig. 2A). After initial quality control, we obtained a single-cell transcriptome from 62,788 cells in the ED samples and 19,766 cells in the non-ED samples (Table S1). To explore the cellular composition of ED samples, we conducted PCA to study gene expression variability among cells and identified nine major clusters, including EC, ECs_lympho, FBs, Mono, Schwann cells, neutrophils, regulatory T cells, SMCs, and T cells (Fig. S1A-D). The percentage of each cell type in each sample is shown in Fig. S1E. We found that the ECs_lympho and regulatory T cells were not exist in three non-ED groups (Fig.S1E). To compare the differences between ED and non ED, we included the remaining seven cell types in the analysis (Fig. 2B–C). We then performed differential gene expression analysis to generate cluster-specific marker genes to determine the identity of each cell cluster and generated GO enrichment analysis on the differential expression genes (DEGs) of clustered cell types. Notably, we found that the enrichment results for the first five cells included ECM (Fig. 2D). In most cases, well-known cell type markers are used to identify cell clusters, such as VWF and PECAM1 in vascular ECs, and TAGLN and ACTA2 in SMCs (Fig. S1C). Because the changes in molecular phenotype provide insights into the functional changes of single-cell types, we functionally annotated the ED-related DEGs in single cells by performing pathway analysis (Table S2). Our findings demonstrated that the DEGs in FBs, ECs, Schwann cells, Mono, and neutrophils (e.g., *COL1A1*) were associated with ECM organization. In addition, our pathway analysis also revealed that the DEGs in FBs (e.g., *IGF1*, *COL1A1*, *DCN*, *COL1A2*, *CFD*), ECs (e.g., *PLAT*, *ADGRF5*, *MMRN1*, *EMCN*), and Schwann cells (e.g., *APOD* and *GPC3*) were associated with the regulation of vasculature development, cell-substrate, and negative regulation of locomotion, respectively (Fig. 2E).

### Disruption of FBs subpopulation heterogeneity contributes to change in ECM in ED

**Figure 3:**
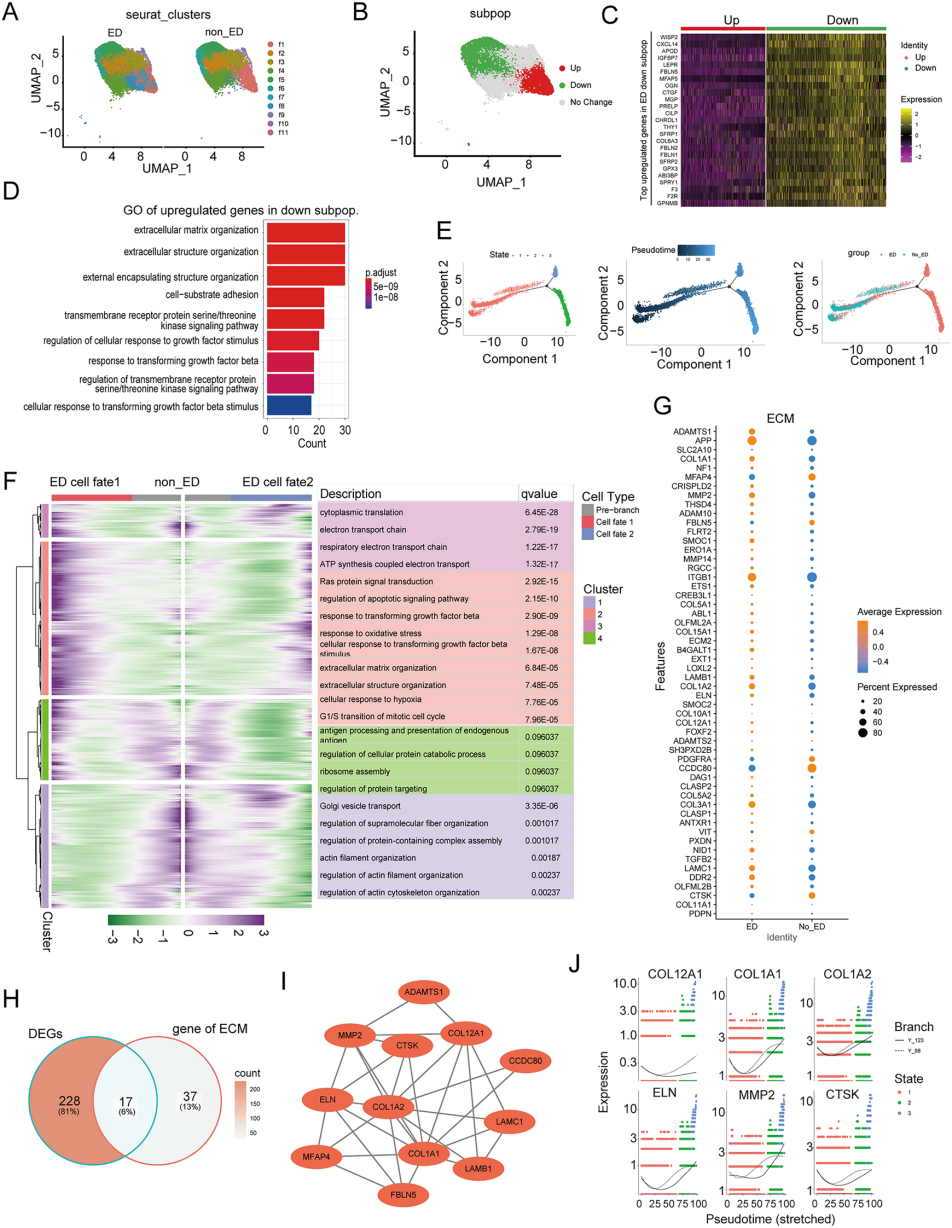
The Disrupted Subpopulation Heterogeneity of fibroblast Contributes to ECM alteration in ED. (**A**) UMAP plots showing the distributions of FBs subpopulations (FBs1–FBs11) in ED and NC corpus cavernosum samples. (**B**–**D**) The FBs subpopulation associated with the organization of ECM functions was reduced in ED. (**B**) UMAP plot showing the distribution of t he ED-associated FBs subpopulations. (**C**) Heatmap showing the expression levels of the top enriched genes in the ED-down-regulated FBs subpopulation (adjusted *P* < 0.05, log2 fold change ≥ 0.5). Down: down-regulated subpopulation; Up: up-regulated subpopulation. (**D**) GO pathway analysis of the transcriptomic signature of the ED-down-regulated FBs subpopulation. (**E**) The developmental trajectory of FBs in each cluster inferred by Monocle2, each point corresponds to a single cell. From left to right, representing different states, different times and different groups. (**F**) Heatmap showing that the differentially expressed genes (rows) along the pseudo-time (columns) is clustered hierarchically into four profiles. The representative gene functions and pathways of each profile were shown. (**G**) Dot plot represents the differential expression of ECM-related genes filtering from ECM organization pathways. (**H**) Veen graph intersected the down-regulated genes and ECM-related pathways-related genes. (**I**) The PPI network of ECM-related in FBs of the corpus cavernosum between ED and non-ED. (**J**) The scatter plot of gene expression of different types of cells along the pseudo-time trajectory of different hub genes.

Next, we studied the subgroup heterogeneity of single-cell types in the ED and non-ED samples by conducting a subgroup analysis of the single-cell types. FBs subpopulation analysis identified 11 distinct subpopulations of the transcriptome (Fig. 3A and Fig. S2A,B). The relative proportions of the subpopulations f1, f4, f8, f9, f10, and f11 were similar among the ED and non-ED samples (Fig. 3B, Fig. S2C, and Tables S3-4). However, only subgroups f2, f3, f5, f6, and f7 expressed DEGs; Specifically, the f2 and f7 subgroups were enriched in upregulated characteristic genes, whereas the f3, f5, and f6 subgroups were enriched in downregulated characteristic genes (Fig. S2D). Therefore, we categorized f2 and f7 as the “ED-upregulated” subpopulations, and f3, f5, and f6 as the “ED-downregulated” subpopulations (Fig. 3B).

The transcriptome of the ED-downregulated FBs subpopulations was characterized by enriched expression of genes associated with ECM organization, including *FBLN5*, *SFRP2*, and *COL4A1* (Fig. 3C,D and Tables S5-6). In previous studies, impaired ECM organization resulted in ED^13–15^. In contrast, ED-upregulated subpopulations exhibited enriched expression of FBs proliferation-associated genes, including *AGT*, *AQP1*, *JUN*, and *CDKN1A*, as well as the *ERK1/2* cascade (Fig. S2E and Table S7). Taken together, our results showed that in ED, (i) five specific FBs subpopulations exhibit transcriptomic changes, (ii) ECM organization is impaired (f2 and f7), and (iii) the ERK1/2 cascade-associated FBs proliferation is induced (f3, f5, and f6).

Furthermore, we analyzed the expression patterns of all FBs genes along the trajectory of ED and identified 27,449 genes with dynamic expression changes (Fig. 3E). We further classified these genes into two ED clusters (Cluster1-2) and two non-ED clusters (Cluster3-4) according to their expression patterns. Functional enrichment analysis showed that the expression levels of genes involved in the regulation of supramolecular fiber organization, actin filament organization, and actin cytoskeleton organization were higher in FBs in the non-ED state. In contrast, we identified multiple classical processes that were activated during ED progression, including ECM organization and extracellular structure organization. In particular, a large portion of the genes responsible for the apoptotic signaling pathway, response to transforming growth factor beta (*TGF-β*), and oxidative stress were remarkably activated during ED progression (Fig. 3F and Table S8). We also detected the activation of ECM-associated genes that contribute to the progression of ED (Fig. 3G and Table S8). Based on DEGs in the FBs subpopulation analysis and ECM-related genes, we identified 30 ECM-associated DEGs (Fig. 3H) and considered *COL1A1* and *COL1A2* as the hub genes of ECM (Fig. 3I). In addition, The scatter plot of gene expression of different types of cells along the pseudo-time trajectory of different hub genes (*COL12A1, COL1A1, COL1A2, ELN, MMP2,* and *CTSK*) (Fig. 3J).

### Disrupted subpopulation heterogeneity of SMCs contributes to ECM alteration in ED

**Figure 4:**
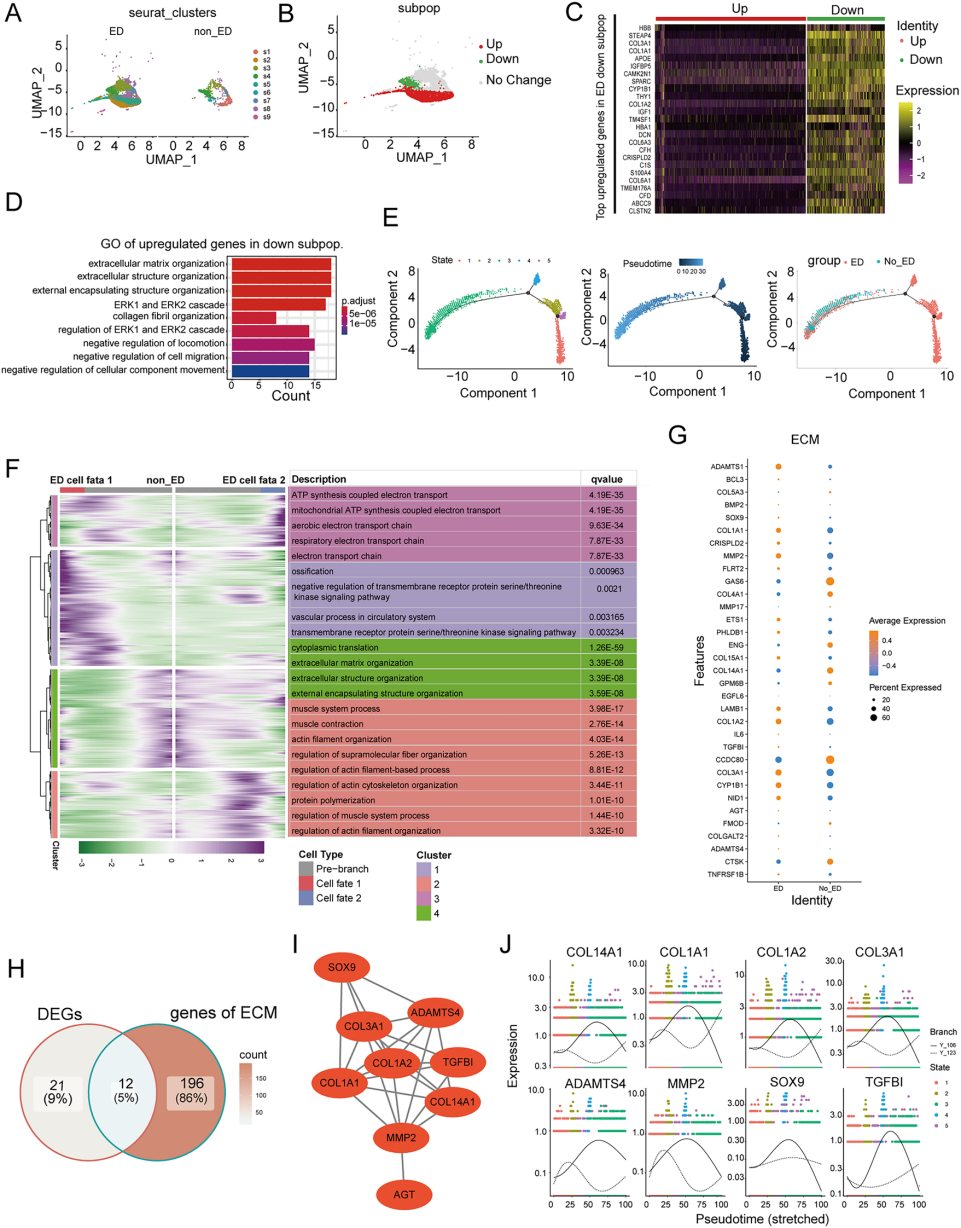
The Disrupted Subpopulation Heterogeneity of smooth muscle cells Contributes to ECM alteration in ED. (**A**) UMAP plots showing the distributions of FBs subpopulations (SMCs1–SMCs9) in ED and NC corpus cavernosum samples. (**B**–**D**) The SMCs subpopulation associated with the organization of ECM functions was reduced in ED. (**B**) UMAP plot showing the distribution of the ED-associated SMCs subpopulations. (**C**) Heatmap showing the expression levels of the top enriched genes in the ED-down-regulated FBs subpopulation (adjusted *P* < 0.05, log2 fold change ≥ 0.5). Down: down-regulated subpopulation; Up: up-regulated subpopulation. (**D**) GO pathway analysis of the transcriptomic signature of the ED-down-regulated SMCs subpopulation. (**E**) The developmental trajectory of SMCs in each cluster inferred by Monocle2, each point corresponds to a single cell. From left to right, representing different states, different times and different groups. (**F**) Heatmap showing that the differentially expressed genes (rows) along the pseudo-time (columns) is clustered hierarchically into four profiles. The representative gene functions and pathways of each profile were shown. (**G**) Dot plot represents the differential expression of ECM-related genes filtering from ECM organization pathways. (**H**) Venn diagram intersected the down-regulated genes and ECM-related pathways-related genes. (**I**) The PPI network of ECM-related genes in SMCs of the corpus cavernosum between ED and non-ED. (**J**) The scatter plot of gene expression of different types of cells along the pseudo-time trajectory of different hub genes.

We then performed a subcluster analysis of the SMCs, which revealed eight subpopulations with distinct transcriptome profiles (Fig. 4A and Fig. S3A,B). The relative proportions of subpopulations s1, s4, and s6 were similar among ED and non-ED samples (Fig. S3C). However, only subpopulations s2, s3, and s5 expressed DEGs; specifically, subpopulations s2 and s5 were enriched in upregulated signature genes, whereas subpopulation s3 was enriched in downregulated signature genes (Fig. S3D and Tables S9-10). Therefore, we categorized s2 and s5 as the “ED-upregulated” subpopulations, and s3 as the “ED-downregulated” subpopulation (Fig. 4B).

The transcriptome profile of the ED-downregulated SMCs subpopulation was characterized by the enriched expression of genes associated with ECM organization, including *COL3A1*, *COL1A1*, and *CYP1B1* (Fig. 4C,D and Tables S11, 12). Previous studies suggested that impaired ECM organization results in ED^13–15^. The ED-upregulated subpopulations exhibited enriched expression of genes associated with the muscle system process, including *LMOD1*, *SMTN*, *FBXO32*, *MYL9*, and *FLNA*, and elastic fiber assembly, including *MFAP4*, *THSD4*, and *MYH11* (Fig. S3E and Table S13).

Furthermore, we analyzed the expression patterns of all SMCs genes along the trajectory of ED, and identified 3867 genes with dynamic expression changes (Fig. 4E). We further classified these genes into two ED clusters (Cluster1-2) and two non-ED clusters (Cluster3-4) according to their expression patterns (Fig. 4F). Functional enrichment analysis showed that genes with an electron transport chain have higher expression levels in SMCs in the ED state than in the non-ED state (Table S14). Additionally, ECM organization, extracellular structure organization, and external encapsulating structure organization were activated during ED progression, consistent with the findings of the SMCs subpopulation analysis (Fig. 4D). We also detected regulation of muscle system process-associated genes that participate in ED progression (Fig. 4F and Table S15). Then, based on the DEGs in the SMCs subpopulation analysis and ECM-related genes (Fig. 4G), we identified 12 ECM-associated DEGs (Fig. 4H), and considered *COL3A1*, *COL14A1*, and *COL1A2* as the hub genes of ECM (Fig. 4I). In addition, The scatter plot of gene expression of different types of cells along the pseudo-time trajectory of different hub genes (Fig. 4J).

### Disrupted subpopulation heterogeneity of ECs contributes to endothelial dysfunction in ED

**Figure 5:**
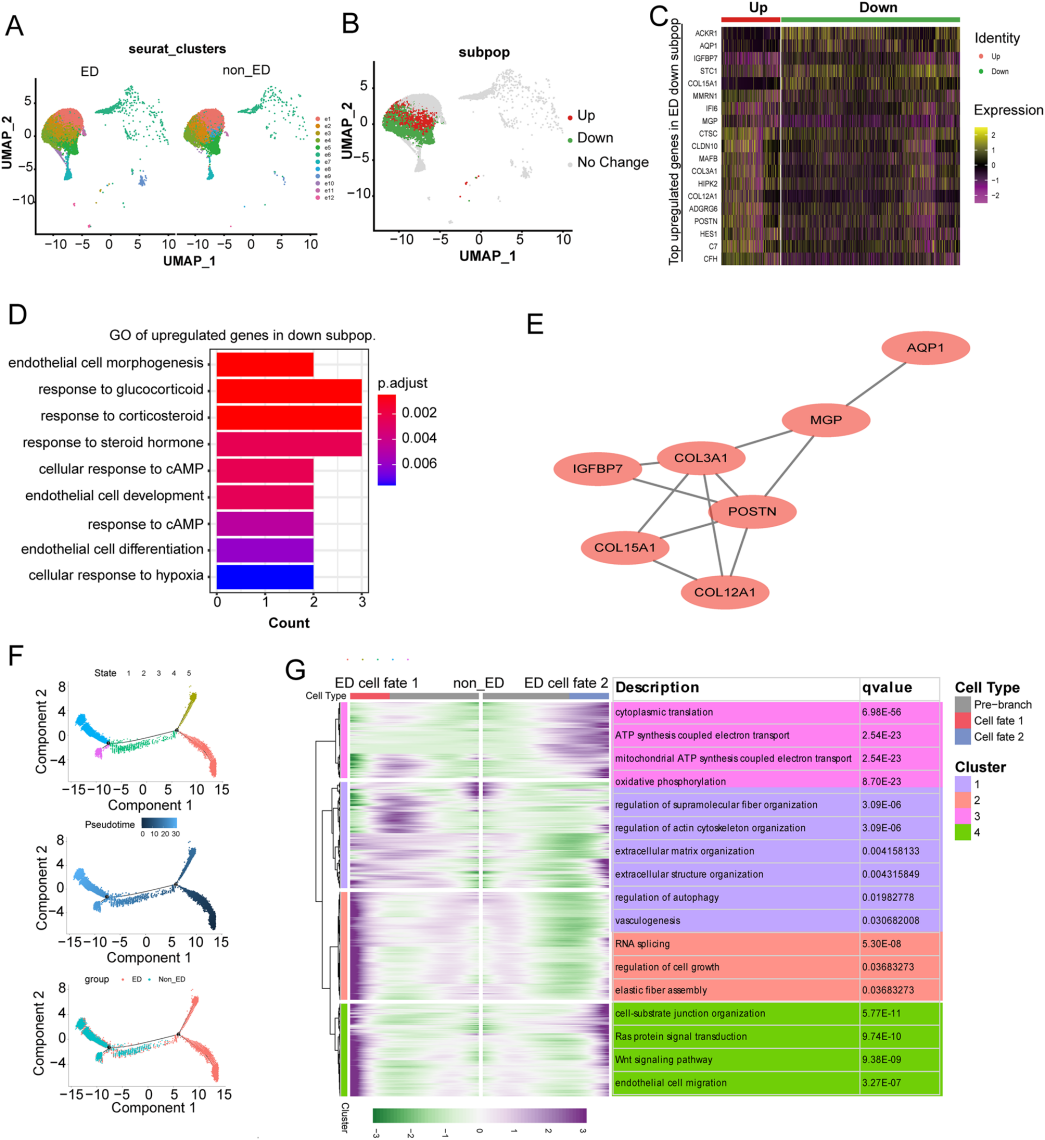
The Disrupted Subpopulation Heterogeneity of endothelial cells Contributes to ECs alteration in ED. (**A**) UMAP plots showing the distributions of ECs subpopulations (ECs1–ECs12) in ED and NC corpus cavernosum samples. (**B**–**D**) The ECs subpopulation associated with endothelial cell morphogenesis was reduced in ED. (**B**) UMAP plot showing the distribution of the ED-associated ECs subpopulations. (**C**) Heatmap showing the expression levels of the top enriched genes in the ED-down-regulated ECs subpopulation (adjusted *P* < 0.05, log2 fold change ≥ 0.5). Down: down-regulated subpopulation; Up: up-regulated subpopulation. (**D**) GO pathway analysis of the transcriptomic signature of the ED-down-regulated ECs subpopulation. (**E**) The PPI network of the top enriched genes. (**F**) The developmental trajectory of ECs in each cluster inferred by Monocle2, each point corresponds to a single cell. From left to right, representing different states, different times and different groups. (**G**) Heatmap showing that the differentially expressed genes (rows) along the pseudo-time (columns) is clustered hierarchically into four profiles. The representative gene functions and pathways of each profile were shown.

Thereafter, we performed a subcluster analysis of ECs, which identified eight subpopulations with distinct transcriptome profiles (Fig. 5A and Fig. S4A,B). The relative proportions of subpopulations e1, e5, e6, e7, e8, and e9 were similar among ED and non-ED samples (Fig. S4A,C, and Table S16, 17). However, only subpopulations e2, e3, and e4 expressed DEGs; specifically, subpopulation e3 were enriched in upregulated signature genes, whereas subpopulations e2 and e4 were enriched in downregulated signature genes (Fig. S4B,D). Therefore, we considered e3 as the “ED-upregulated” subpopulation, and e2 and e4 as the “ED-downregulated” subpopulations (Fig. 5B). The transcriptome profile of the ED-downregulated ECs subpopulation was characterized by enriched expression of genes associated with cellular response to cyclic adenosine monophosphate (cAMP) and hypoxia, including *AQP2* and *STC1* (Fig. 5C,D and Table S18-19). The second-messenger molecules cAMP and cGMP are important endogenous mediators of many cellular processes and are broken down by phosphodiesterase (PDE) to modulate smooth muscle motility. PDE inhibitors block the breakdown of these molecules and consequently elevate the intracellular levels of cAMP, cGMP, or both^16^. PPI co-expression of DEGs showed that *COL3A1* and *POSTN* could be further studied as hub genes (Fig. 5E).

Furthermore, we analyzed the expression patterns of all genes in ECs along the trajectory of ED progression (Fig. 5F and Table S20). We further classified these genes into two ED clusters (Cluster1-2) and two non-ED clusters (Cluster3-4) according to their expression patterns. Functional enrichment analysis showed that genes that regulate oxidative phosphorylation in ECs had higher expression levels in the ED state than in the non-ED state. Functional enrichment analysis also identified genes involved in ECM organization, regulation of autophagy, vasculogenesis, regulation of cell growth, and the Wnt-signaling pathway (Fig. 5G).

### Comparing intracellular communication between FBs and other cells in ED and non-ED

**Figure 6:**
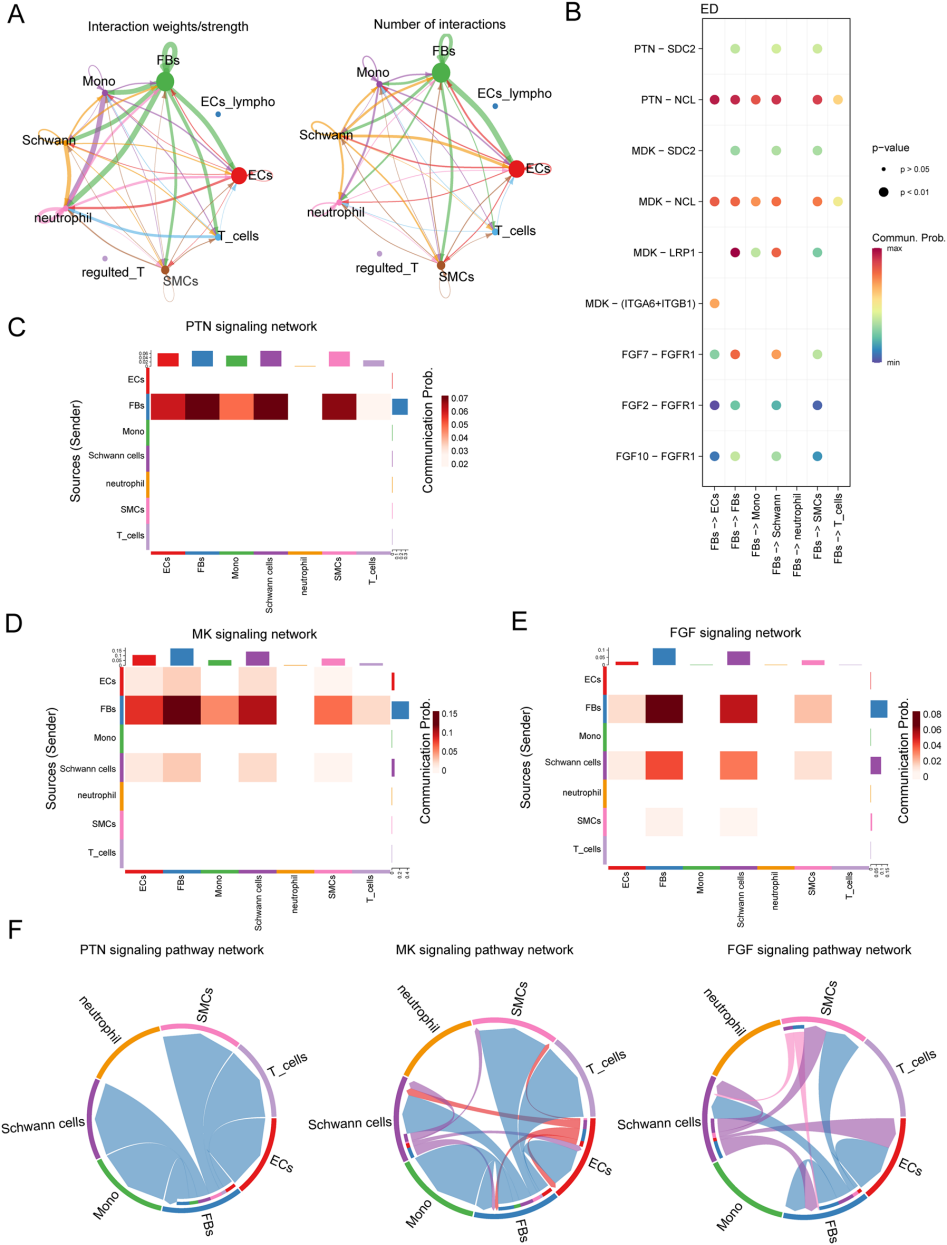
Intracellular analysis ligands sourced from FBs and mainly signal pathways in ED samples. (**A**) Net plot showing the interaction number and strength. (**B**) Network plot showing the specific communicating pathways and their strength. (**C**, **D**, **E**) Heatmap showing the communication interaction of PTN, MK and FGF pathways in ED. The communication probability of a signaling pathway was computed by summarizing the probabilities of its associated ligand–receptor pairs. The darker the color, the greater the communication probability between the two cell types. (**F**) Chord diagram showing the cell–cell communication interaction of PTN, MK and FGF pathways in ED.

Next, we investigated the interaction between all cells, especially FBs, ECs, and SMCs, in patients with and without ED. For the comparison, we first analyzed the cellular interactions in the ED. The net plot shows that FBs accounted for most interactions among all cell types. Except for the cell-in-cell interactions among FBs, ECs mostly interacted with FBs, and to a lesser extent with Schwann cells and SMCs (Fig. 6A and Fig. S5A). A detailed cell–cell interaction network showed that FBs contributed to signaling pathways via various ligand-receptor interactions, among which the *PTN-SDC2, PTN-NCL, MDK-SDC2, MDK-NCL, MDK-LRP1, FGF7-FGFR1, FGF2-FGFR1,* and *FGF10-FGFR1* signaling pathways were the most prominent (Fig. 6B). The heatmap of the PTN signaling pathway showed a strong inter-association between FBs and Schwann cells, secondly between FBs and SMCs, as shown in Fig. 6C. The heatmap of the MK signaling pathway showed a strong inter-association between FBs and Schwann cells, secondly FBs and ECs, as shown in Fig. 6D. The heatmap of the *FGF* signaling pathway showed a strong inter-association between FBs and Schwann cells, as shown in Fig. 6E. These cellular activities were most likely influenced by FBs, followed by Schwann cells, SMCs, and ECs (Fig. 6F). The neurons represented by Schwann cells may be closely related to the extracellular matrix represented by fibroblasts, which is also the key factor causing ED.

**Figure 7:**
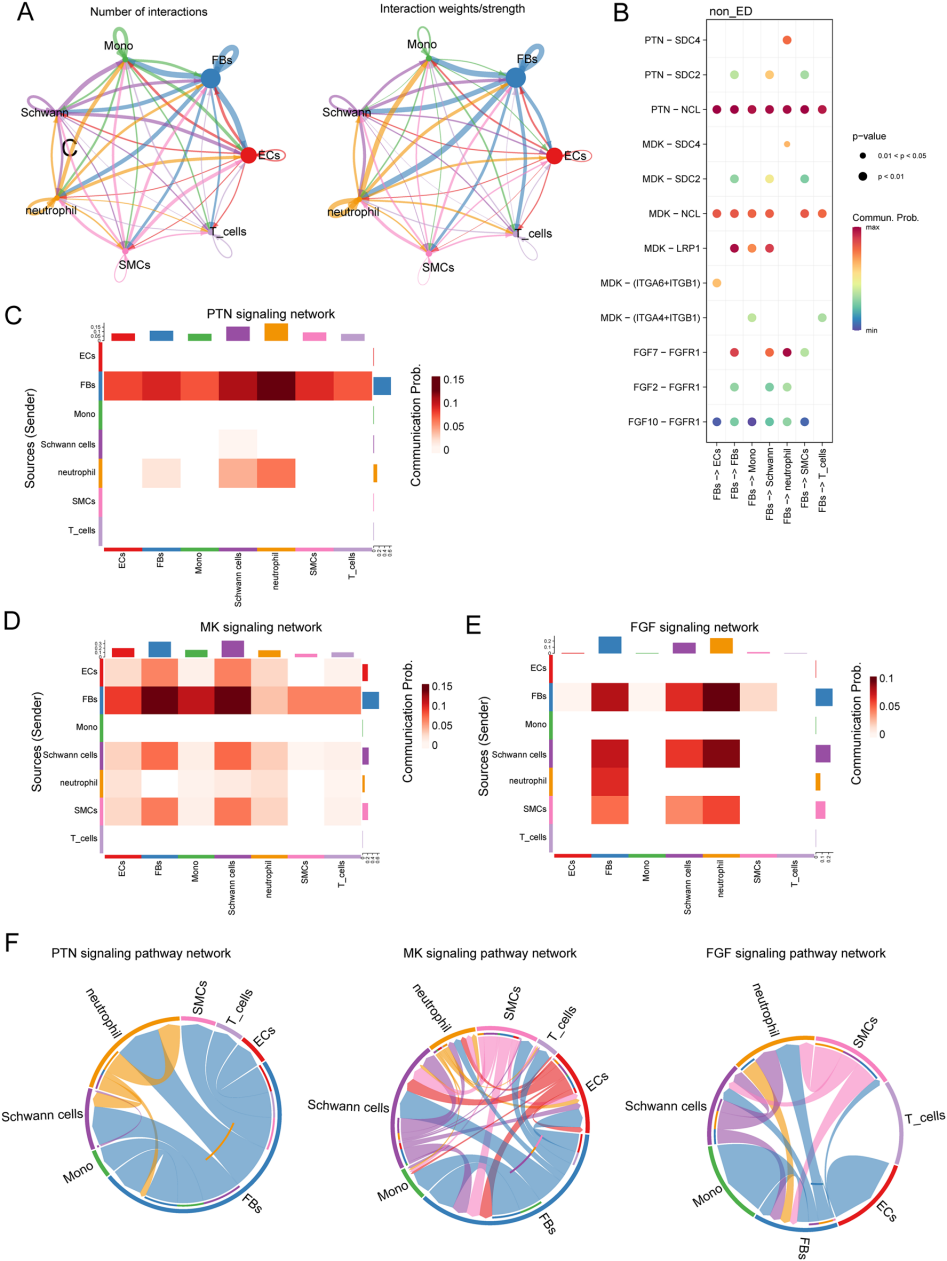
Intracellular analysis ligands sourced from FBs and mainly signal pathways in non-ED samples. (**A**) Net plot showing the interaction number and strength. (**B**) Network plot showing the specific communicating pathways and their strength. (**C**, **D**, **E**) Heatmap showing the communication interaction of PTN, MK and FGF pathways in non-ED. The communication probability of a signaling pathway was computed by summarizing the probabilities of its associated ligand–receptor pairs. The darker the color, the greater the communication probability between the two cell types. (**F**) Chord diagram showing the cell–cell communication interaction of PTN, MK and FGF pathways in non-ED.

A parallel cell–cell interaction analysis was performed for the control group (non-ED group). Owing to the fewer cell types and a lack of regulatory T cells and endothelial lymphocytes, the net plot exhibited less complex intracellular interactions in the non-ED group (Fig. 7A). Notably, the leading communication signaling pathways in the non-ED group were still the *PTN, MK,* and *FGF* pathways, but partly interacted with each cell cluster compared with ED. *MDK-LRP1* and *FGF2-FGFR1* interactions between FBs and SMCs were also exclusively observed in the ED rather than in the non-ED. Furthermore, the *PTN-SDC2* and *MDK-SDC2* pathways from FBs contributed more to the interaction with SMCs in the ED rather than in the non-ED group. Additionally, *MDK-(ITGA4-ITGB1)* and *PTN-SDC4* pathways were decreased in ED group as compared to that in non-ED group (Figs. 6B, 7B).

Additionally, the heatmap of the *PTN* signaling pathway showed a strong inter-association between FBs and neutrophil cells, as shown in Fig. 6C. Heatmap of the *MK* signaling pathway showed a strong inter-association between FBs and Schwann cells, as shown in Fig. 7D. Heatmap of the *FGF* signaling pathway showed a strong inter-association between FBs and neutrophil cells, as shown in Fig. 7E.

Intriguingly, the cell–cell communication in normal group represents more complex (Figs. 6F, 7F), This implies that cell–cell interactions receive inhibition in ED patients as compared with normal patients.

### Alteration of ECM regulatory genes in bilateral corpus cavernosum injury (BCNI) model rat

**Figure 8:**
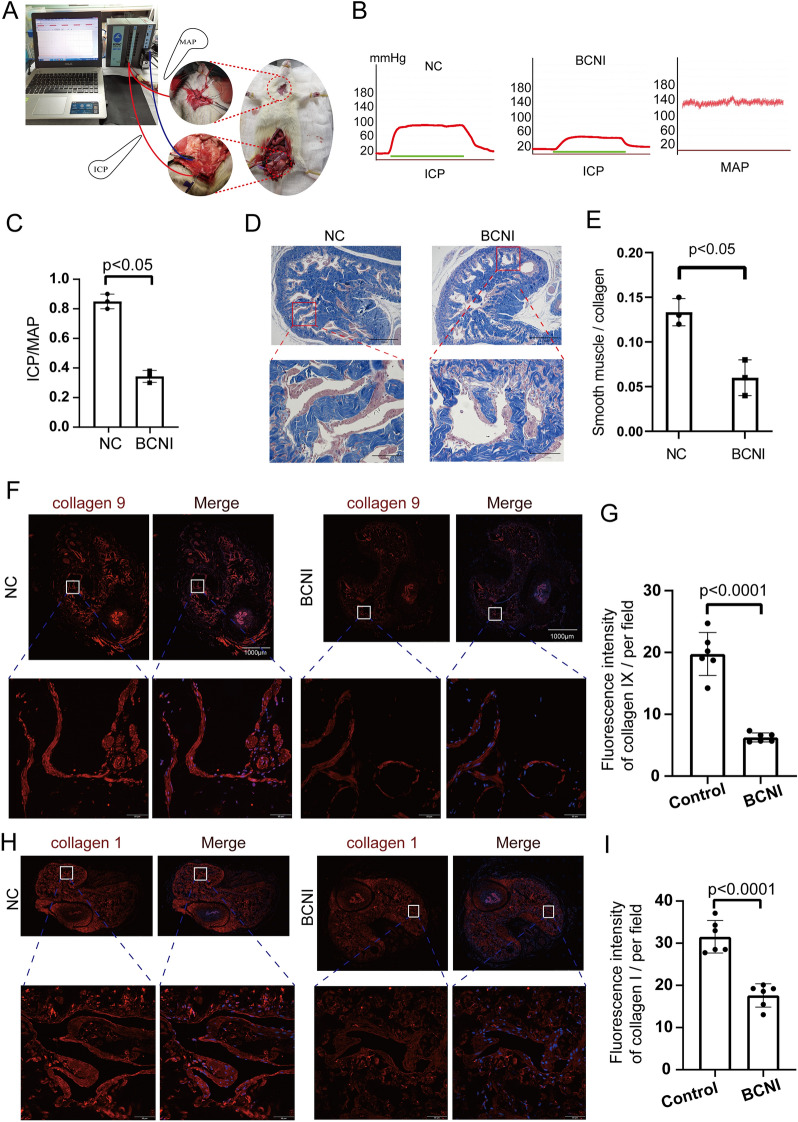
Constructing an erectile dysfunction model in SD rats with bilateral cavernous nerve injury (BCNI) and ECM-related protein expression level expression of cavernous tissue in rats with BCNI. (**A**, **B**) The representative ICP and MAP of each experimental group were recorded under the following conditions: model: low voltage, method: continuous single stimulation, frequency: 20 Hz, intensity: 5 V. The stimulus interval indicated by the solid bar. Erectile function, as determined in vivo by electrical stimulation of the cavernous nerve of the rats in Sham group and BCNI group. (**C**) The erectile function of each group is presented as the maximal ICP/MAP ratio in the bar graph. (**D**) Masson staining of cavernous tissue sections performed Smooth muscle/Collagen ratio in the Sham group and BCNI group. (**E**) The ratio was quantified in the bar graph. (4 × magnification, upper row; 20 × magnification, lower row). Lower rows represent enhanced magnification of white rectangle boxes in upper rows. (**F**–**I**) Immunofluorescence staining of cavernous tissue sections performed with anti- collagen 1, anti-collagen 9 antibody in the Sham group and BCNI group (10 × whole tissue cross-sectional scan, upper row; 40 × magnification, lower row). Lower rows represent enhanced magnification of red rectangle boxes in upper rows. And staining intensity was quantified and presented as fluorescence intensity. Values are mean ± SD (n = 6 per group). #*p* < 0.05 compared with the BCNI group; *p < 0.05 compared with the Sham group.

**Figure 9:**
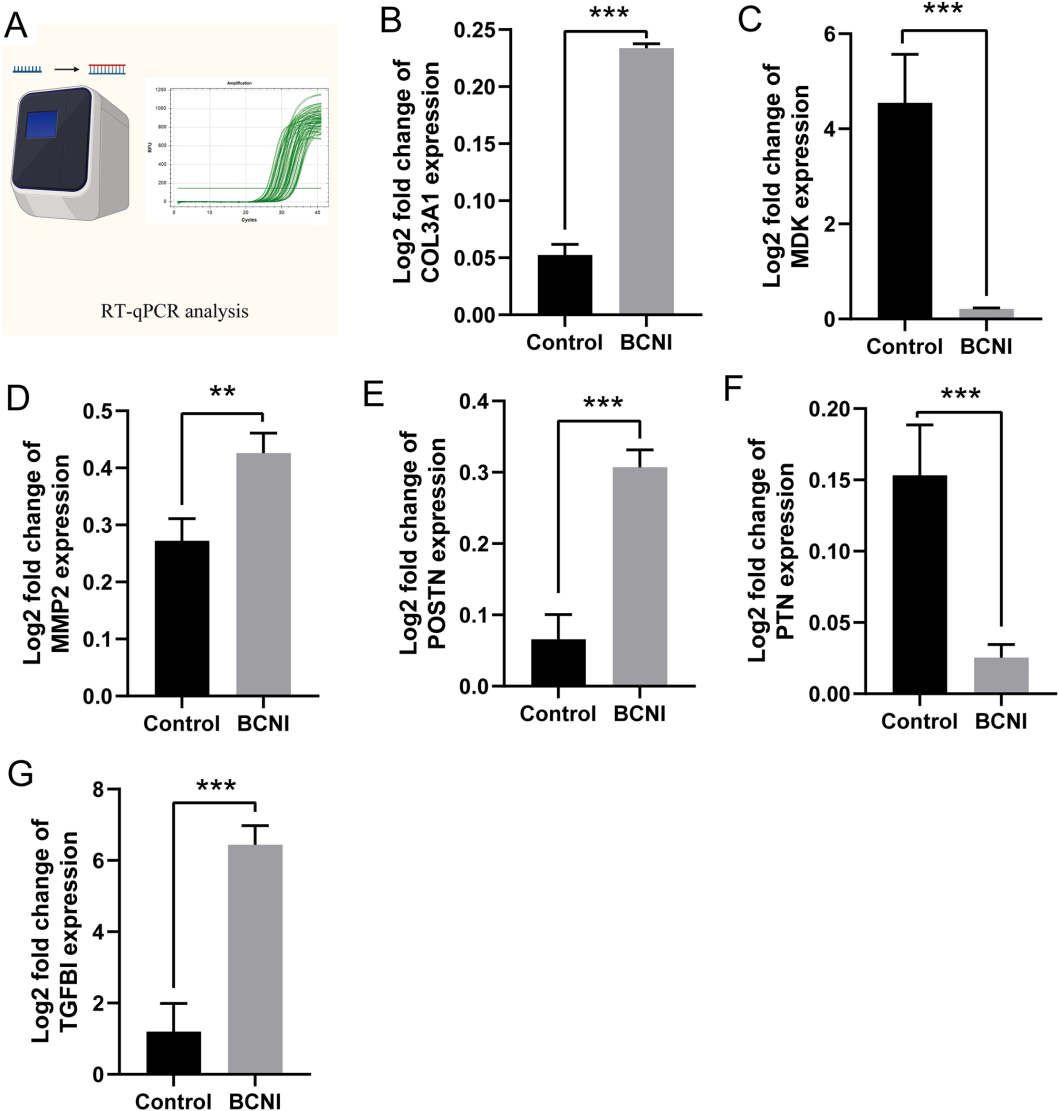
Identification of key genes with BCNI rat model by RT-PCR. The expression of COL3A1, TGFBI, MMP2, POSTN, PTN, and MDK. (ns, No significancy, *p < 0.05, **p < 0.01, ***p < 0.001).

To pinpoint the genes regulating the ECM, we developed a rat model of ED using bilateral cavernous nerve injury (BCNI) (Fig. 8A–C). Recognizing that collagen, elastin, proteoglycans, and glycosaminoglycans are central to ECM composition, we assessed the smooth muscle-to-collagen ratio in the corpus cavernosum of the penis. Our findings revealed a notable decrease in this ratio in the BCNI group compared to the control group (Fig. 8D,E). Furthermore, the expression levels of ECM-related proteins, such as collagen 1 and collagen 9, were decreased in the BCNI group than in the control group (Fig. 8F–I). Additionally, in comparison to the control rat model, the expression levels of COL1A1, COL3A1, TGFBI, MMP2, POSTN, PTN, and MDK in ED rats were significantly decreased (Fig. 9A–G). This suggests that there have indeed been changes in ECM regulatory genes in the ED model rats.

## Discussion

Identifying precise molecular and cellular targets for the development of ED treatment requires a comprehensive understanding of the cell type specific response and cell heterogeneity of ED. Accordingly, our single-nucleus transcriptome analysis identified the three major molecular pathways that are extracellular matrix organization in fibroblasts, SMC, ECs, and T cells; regulation of vasculature development, vasculature development, cell-substrate in fibroblasts; and negative regulation of locomotion in Schwann cells. In addition, we found that the dysregulation of these pathways in ED is due to changes in the heterogeneity of subpopulations of corresponding cell types. Interestingly, we have found that some hub genes may regulate ECM, affecting various specific cellular functions, such as FBs, SMCs, and ECs. ECM has been proven to be very important for ED in animal experiments, human studies on the role of ECM in ED are very rare. It is of great significance to study the relationship between ECM and various subgroups of cells in cavernous tissue.

Understanding the precise molecular and cellular targets for developing ED treatments necessitates an in-depth knowledge of cell type-specific responses and cellular heterogeneity in ED. Our single-nucleus transcriptome analysis identified three key molecular pathways: ECM organization in fibroblasts, smooth muscle cells (SMCs), endothelial cells (ECs), and T cells; regulation of vasculature development and cell-substrate interaction in fibroblasts; and the negative regulation of locomotion in Schwann cells. Additionally, our findings suggest that the dysregulation of these pathways in ED may be attributed to alterations in the heterogeneity of subpopulations within these cell types. Intriguingly, we discovered that certain hub genes potentially regulate the ECM, impacting specific cellular functions of fibroblasts (FBs), SMCs, and ECs. Although ECM's importance in ED has been established through animal studies^17,18^, human research exploring the role of ECM in ED is sparse. Investigating the interaction between ECM and various cellular subgroups in cavernous tissue is therefore of paramount importance.

Heterogeneity of fibroblasts is a marker of age-related erectile dysfunction^19^. Patients with ED show obvious fibrosis of the cavernous tissue. Fibrosis is a pathological process that occurs in many diseases. Its main feature is the excessive accumulation of ECM, leading to chronic inflammation. Fibrosis can be an abnormal (or excessive) wound-healing reaction that usually occurs as the final pathological result of chronic tissue injury^20^. However, the exact trigger of the fibrotic process remains unknown. In FBs, we found that the *ERK1/2* cascade and ECM organization are altered in ED group. This is consistent with previous studies^21^, that is, active *ERK1/2* in patients with ED is significantly increased, which has an inhibitory effect on cavernosal *eNOS* activity, an important factor in penile erection. To demonstrate this result, we also find changes in the ECM organization and related DEGs, such as *COL12A1* and *COL1A1*. Previous research has demonstrated that the upregulation of genes encoding type I collagen (*COL1A1* and *COL1A2*) enhances the secretion of ECM components, including proteoglycan and type I collagen^22^. *COL1A1* plays a critical role in the synthesis, proliferation, and migration of ECM in human skin FBs following thermal injury^23^. Moreover, it is implicated in various fibrotic conditions such as cardiac, renal, hepatic, and pulmonary fibrosis^24–27^. Consequently, *COL1A1* holds significant clinical potential in the treatment of penile fibrosis.

Peripheral erection control is governed by neuronal activity and local factors, which ultimately influence the contraction or relaxation of the smooth muscle in the corpus cavernosum. Our analysis revealed that in SMCs, not only are pathways related to muscle system processes and muscle contraction activated, but also pathways associated with ECM organization and extracellular structure organization are notably enriched. This enrichment was observed in both the SMCs subgroup analysis and the pseudo-time analysis, mirroring the findings in FBs analysis.

Research has established that a key factor in the development of arteriosclerosis is the role of ECM proteins in supporting mechanical load. This is closely followed by the contribution of vascular smooth muscle cells, which regulate actin interaction during contraction and mediate the mechanical transduction crucial for ECM homeostasis. Furthermore, the plasticity and signal transduction of vascular SMCs in both conductive and resistance arteries play a vital role in the physiology of normal and early vascular aging^28^. This finding underscores the significant role of the ECM in regulating the function of SMCs. Additionally, the aortic wall consists of highly dynamic cell populations and ECM components. In reaction to biomechanical environmental changes, both aortic cells and ECM adapt their structures and functions to enhance the aortic wall's strength, thereby meeting hemodynamic demands^28^. Crucially, our study identifies alterations in the ECM organization and related differentially expressed genes (DEGs), including COL3A1, TGFBI, and MMP2. Among these, COL3A1 is particularly noteworthy for its contribution to ECM changes in airway SMCs during the pathogenesis of asthma^29^. Activating *MMP2* expression can promote the proliferation and migration of human aortic SMCs, and lead to the degradation of surrounding ECM^30^. Furthermore, transforming growth factor-beta-induced protein ig-h3 (TGFBI), an ECM glycoprotein, has not yet been reported in SMCs. However, its potential relevance in the context of ECM and SMCs research remains a promising area for future investigation.

The loss of endothelial functional integrity and the ensuing endothelial dysfunction are pivotal in the development of ED. The interplay between cells and the ECM significantly influences endothelial cell behavior, particularly during angiogenesis^31^. In the ECs subgroup analysis, COL3A1 has been found to have a strong association with endothelial fibrosis^32^. Periostin (POSTN), an ECM protein, is involved in tissue remodeling post-injury. Overexpression of POSTN stimulates hypoxia-inducible factor (HIF) activation and escalates the production of endothelin-1 (ET-1) and vascular endothelial growth factor (VEGF) in human pulmonary artery endothelial cells^33^. Consequently, these findings indicate that COL1A1, COL3A1, and POSTN could potentially be used as biomarkers to assess the severity of endothelial dysfunction in the progression of ED.

Given the research potential of COL3A1 in SMCs and ECs, and MMP2 in FBs and SMCs, we constructed an ED rat model with bilateral corpus cavernosum injury (BCNI). We detected that the ratio of smooth muscle/collagen was decreased in BCNI group as compared with normal group, and higher levels of ECM-related protein expression (collagen1, and collagen9) in normal group than those in BCNI group (Fig. 7F–I). In addition, compared with the normal rat model, the expression levels of COL1A1, COL3A1, TGFBI, MMP2, POSTN, PTN, and MDK in ED rats were significantly decreased. It indicates that the ECM in the ED model rats has indeed undergone alteration.

Considering the significant research potential of COL3A1 in SMCs and ECs, as well as MMP2 in FBs and SMCs, we developed a rat model of ED featuring bilateral corpus cavernosum injury (BCNI) (Fig. 7A–C). In this model, we observed a reduced smooth muscle-to-collagen ratio in the BCNI group compared to the control group, alongside elevated levels of ECM-related proteins (such as collagen1 and collagen9) in the control group compared to the BCNI group (Fig. 7F–I). Furthermore, the changes of key genes suggests that there has indeed been a substantial alteration in the ECM within the ED model rats.

Additionally, our study revealed an increase in the population of regulatory T cells (Treg cells) and lymphatic endothelial cells (LECs) within the ED group. Treg cells, a specific subset of T cells, are known to be essential in maintaining self-tolerance and mitigating inflammatory collateral damage. They achieve this by suppressing overactive (self-reactive) immune responses^34^. Treg cells have been identified in various tissues, including non-lymphoid tissues, where they exhibit tissue-specific functions. These cells play a crucial role in maintaining tissue homeostasis and facilitating tissue repair^35^. Furthermore, LECs are pivotal in the immune response, regulating immune transport, supporting immune cell survival, and presenting antigens to dendritic cells. Consequently, we hypothesize that the increase in Treg cells and LECs observed in ED patients may be attributed to an augmented autoimmune function within the cavernous tissue. This response potentially serves to protect functional cells from damage caused by external stimuli. Such insights could pave the way for new directions in ED treatment.

In this study, the *PTN-SDC2* and *MDK-SDC2* pathways were associated with ED via cell–cell communication. Pleiotrophin (*PTN*) is a secreted growth factor and cytokine associated with the ECM. In recent years, as an important nerve regulator during development, *PTN* received particular attention from researchers. *PTN* is expressed in many tissues and it mainly regulates cell proliferation, growth, and differentiation through different receptors.

In our research, the PTN-SDC2 and MDK-SDC2 pathways were found to be associated with ED through cell–cell communication. PTN, a secreted growth factor and cytokine linked to the ECM, has garnered considerable attention in recent years, particularly for its role as a vital nerve regulator during development. Expressed in a variety of tissues, PTN primarily influences cell proliferation, growth, and differentiation via diverse receptors^36,37^. Expression analysis in dermal fibroblasts (FBs) of individuals with the condition revealed that two genes, Syndecan-2 (SDC2) and Growth Differentiation Factor 6 (GDF6), located within the duplicated region, contribute to the dysregulation of genes encoding ECM proteins and downstream elements in the Transforming Growth Factor-Beta (TGF-β) pathway^38^. Midkine (MDK), forming a unique family of heparin-binding growth factors alongside Pleiotrophin (PTN)^39^, is a multifunctional protein involved in a range of physiological processes including development, reproduction, and repair. Furthermore, MDK is implicated in neurogenesis, epithelial-mesenchymal interactions, and mesoderm remodeling^39^. Notably, among the receptors of the PTN, MDK, and Fibroblast Growth Factor (FGF) pathways, SDC2 and Fibroblast Growth Factor Receptor 1 (FGFR1) influence non-integrin membrane-ECM interactions similarly to Low-Density Lipoprotein Receptor-Related Protein 1 (LRP1)^40^, mediating the clearance of numerous molecules from the ECM^41^. This mechanism significantly affects ECM organization. Therefore, exploring variations in receptor-ligand interaction intensity could present a promising research avenue for ED.

## Conclusions

In summary, our study underscores that the development of ED is closely associated with genes regulating ECM, and alterations in functional intercellular communication. Key factors in the onset of ED include changes in ligand-receptor interactions and genetic modifications. Furthermore, exploring the role of immune regulation presents a promising avenue for future research in the treatment of ED.

## Methods

### scRNA-seq data collection and quality control

We downloaded the original data from the Gene Expression Omnibus (GEO) database (https://www.ncbi.nlm.nih.gov/geo/; GSE206528) and the National Omics Data Encyclopedia (NODE) database (www.biosino.org/node; OEP002391, OEP002948, and OEP003055), which comprised three healthy non-ED samples and six ED datasets^4,5^. Low-quality cells were excluded according to the following criteria: (a) I criteria set in the function ‘CreateSeuratObject’ included: min.cells = 3, min.features = 50, (b) the percentages of mitochondrial and ribosomal genes were less than 5%, (c) two samples in the GSE206528 database were abandoned because they were diabetic samples. According to these criteria, 82,554 single cells were included in the subsequent analyses. The data are presented in Table S1.

### Sample integration and cell classification

The cell matrix gene of each sample was used to create a Seurat object using the Seurat package of R language. Cells were further filtered based on the following threshold parameters: total number of expressed genes, 800–7000; total UMI count, 0–30,000; and proportion of mitochondrial genes expressed, < 10%. Batch correction was performed using the IntegrateData function in the Seurat package (v 4.1.1) of R language (v4.2.1)^42^, according to the package manual (https://satijalab.org/xplor/v3.1/pbmc3k_tutorial.html).

The Seurat package first processes data from the two datasets separately^4,5^. Then, the two datasets (including 9 samples) were integrated by the ‘Seurat’, ‘patchwork’, ‘tidyverse’, ‘dplyr’, and ‘ggplot2’ packages of R language. After integration, genes were summarized using principal component analysis (PCA) to reduce dimensionality. Next, the first 30 principal components were used as inputs for cell clustering, and the cells were visualized in two-dimensional uniform manifold approximation and projection (UMAP) representations. The FindClusters function in the Seurat package (resolution parameter: 0.5) was used to cluster the cells. We annotated the cell types using typical marker genes (Table 1).

**Table 1:** Marker genes of cellular clusters.

Cluster	Marker genes
ECs	*PECAM1*, *APOLD1*
FBs	*IGF*
SMCs	*ACTA2*, *TAGLN*, and *CNN1*
T_ Cell	*CD3G*, *CD3D*, and *CD3E*
neutrophil cells	*S100A9* and *S100A8*
Monocytes (Mono)	*CD14* and *CD68*
Schwann cells	*ASCL1* and *BMP7*
regulted_T cells	*PDGFRA* and *RGS5*
lymphatic ECs	*COL5A2* and *PRSS23*

### Analysis of scRNA-seq data

For the second round of quality control, we followed the workflow described in the Seurat analysis guide. First, we normalized the filter matrices logarithmically and identified highly variable features of each sample using the FindVariableFeatures function (selection.method = vst, nfeatures = 1000). To integrate all nine samples, we used the FindIntegrationAnchors function with the parameter dims = 1:20 to determine the characteristics of the anchored samples, and used the IntegrateData function with the parameter dims = 1:20 to integrate the dataset with the determined anchor. We then scaled the integration matrix and used the RunPCA function with the parameter npcs = 50 to perform linear dimensionality reduction. We used the JackStrawPlot function to visualize the *P*-value distribution of each principal component, and used the first 20 principal components for graph-based clustering.

We used the FindClusters function with the parameter resolution1 for K-nearest neighbor clustering, and the RunUMAP function with the parameter dims = 1:20 for UMAP clustering, which initially generated 19 cell clusters. We used the FindAllMarkers function with the parameters logfc.Threshold = 0.25 and test.Use = wilcox to determine the DEG in each cell cluster using a Wilcoxon rank sum test. Then, we assigned cell type identity to each cell cluster according to the expression of known cell type markers. Further, we identified additional cell type-specific marker genes using the FindAllMarkers function (parameters: logfc.threshold = 0.25 and test.use = wilcox) and a Wilcoxon rank-sum test. For cell type markers, the statistical significance level was set to an adjusted *P*-value of < 0.1. To further confirm the cell-type specificity of these markers, we compared our data with the results of a single R package, which confirmed the expression patterns of these markers in different cell types in our study.

### Examination of cell type-specific transcriptomic changes

We first stratified the samples according to the initial classification of ED and non_ED samples then compared the transcriptome profiles of single cell types between ED and NC samples by Wilcoxon rank sum test using the FindMarkers function (parameter: logfc.threshold = 0 and test.use = wilcox). The statistical significance level of cell type-specific transcriptome changes was set at adjusted *P* < 0.01 and log2 two-fold changes of ≥ 0.1 or ≤ -0.1.

### Differentially expressed gene calculation and gene enrichment analysis

The Seurat function FindAllMarkers (test.Use = wilcox; min.pct = 0.1; logfc.Threshold = 0.25) was used to identify DEGs according to the normalized UMI counts. Unless otherwise specified, the DEGs in each selected subcluster was calculated based on a comparison between the subcluster and the rest of the dataset.

In addition, to complete the functional analysis of DEG, we used the R package clusterProfiler (v 4.4.4) to perform gene ontology (GO) enrichment analysis^43,44^. The sub-ontology biological process was of particular concern. Statistical significance was set at *P* < 0.05.

### Pseudo-time analysis

The Monocle2 package (v 2.22.0) of R language was used to perform a pseudo-time analysis to detect cell-state transitions^45^. The top 100 differentially expressed genes in the ED and non-ED cavernous cells (ECs, SMCs, and FBs) were identified by Seurat to sort cells in a pseudo-time order. The cells state highlighted the start point of the pseudo-time in the first round of ‘orderCells’. We then set the cells with abnormal gene expression profiles as the root_state argument and called ‘orderCells’ again. ‘DDRTree’ was applied to reduce dimensions and the visualization function ‘plot_cell_trajectory’ was used to plot the minimum spanning tree on cells. DEGs genes over the pseudo-time from cavernous cells with non-ED gene expression profiles to ED were calculated by the “differentialGeneTest” function in Monocle2 (q-value < 10^−4^). The DEGs in each subgroup were used in the ECs, SMCs, and FBs cell trajectory analyses. The DEGs should have an expression threshold of ≥ 0.5 and discrete expression of ≥ 1. Further, the “deviation_Fit” function in Monocle2 was used to classify cells in pseudo-chronological order. To analyze the function of DEGs, enrichment analysis of GO was conducted using the R package clusterProfiler (v 4.2.2).

### Protein–protein interactions (PPI) of DEGs

To analyze the genome-wide PPI of DEGs, the genes were imported into STRING dataset (https://string-db.org), and got a document of ‘tsv’ format. Next, the document was imported into the Cytoscape software, which is used for beautify the interaction network analysis.

### Cell–cell communication analysis

To identify and visualize the cell cell interaction between ED and NC samples, we used the R package CellChat (1.1.3). We conducted CellChat analysis according to the official workflow. All databases were used, including Secretory Signals, ECM Receptors, and Cell Cell Contact. The Network plot and Chord diagram were used to describe the cell–cell communication in signalling pathway.

### Animal experiment design, functional analysis

Ten 8-week-old male Sprague–Dawley (SD) rats, weighing approximately 200–250 g, were provided by the Experimental Animal Center of Guizhou Medical University, and were randomly and equally divided into two groups: (1) sham (CN expose surgery only, no nerve crushing); (2) control (BCNI procedure). The BCNI procedure was reported previously^46^. The animals were cared for and housed under strict guidelines established by Animal Ethics Committee of Guizhou Medical University.

At the end of 4 weeks of modeling, the animals were recorded for intracavernosal pressure (ICP) and the corresponding arterial pressure (AP) with CN stimulation (CNS) under pentobarbital anesthesia^47^. At the completion of functional analysis, the penis was excised for histopathology.

### Immunofluorescence analysis and laser confocal microscope

Tissue slides were prepared according to standard protocol, which was reported previously^48^. After antigen repair, goat serum was inoculated at room temperature for 30 min, the collegen I antibody (ab260043; Abcam) and collegen IX antibody (orb18265; biorbyt) were incubated overnight respectively, and a secondary antibody solution of AlexaFluor549 (1:200, ThermoFisher) was added for 1 h at 37 °C. slides were washed in PBS, pH 7.4. slides were mounted with anti‐fading medium and counterstained with 4′,6‐diamidino‐2‐phenylindole (DAPI) and images were captured using laser confocal microscopy (Olympus, Tokyo, Japan).

### Real-time reverse transcription PCR (QRT-PCR)

Cavernous tissue RNA was extracted and used for qRT-PCR analysis. The primer sequences we used were:

*β*-Actin, sense: 5′-GATCA-AGATCATTGCTCCTCCTG-3′,

anti-sense: 5′-AGGGTGTAAAACGCAG-CTCA-3′;

MDK, sense: 5′-TTCTAGCCCTTGTTGCCCTCTTG-3′,

anti-sense:5′- AACCCATGCCGCAGTCCTTG-3′;

PTN, sense: 5′-ACCGCCTTGAAGACCAGAACTG-3 ′,

anti-sense: 5′- GCTTGGGCTTGGTGAGTTTGC-3′;

POSTN, sense: 5′-CACTTTCACGGACCTGGTAGCC-3′,

anti-sense: 5′-CAGAGTGTCATCAGAGAACGCATTG-3′;

MMP2, sense: 5′-GACGGCTTCCTCTGGTGTTCC-3′,

anti-sense: 5′-AACTTGCAGGGCTGTCCATCTC-3′;

TGFBI, sense: 5′-TTCCAAGCCATGCCTCCAGAAG-3′,

anti-sense: 5′-GAGACTTCAGCCGCACCAGAG-3′;

COL3A1, sense: 5′-TGGTACTTCTGGTCCTCCTGGTC-3′,

anti-sense: 5′- CGCACCGCCTGGCTCAC-3′.

### Supplementary Information

Supplementary Figures.

Supplementary Table S1.

Supplementary Table S2.

Supplementary Table S3.

Supplementary Table S4.

Supplementary Table S5.

Supplementary Table S6.

Supplementary Table S7.

Supplementary Table S8.

Supplementary Table S9.

Supplementary Table S10.

Supplementary Table S11.

Supplementary Table S12.

Supplementary Table S13.

Supplementary Table S14.

Supplementary Table S15.

Supplementary Table S16.

Supplementary Table S17.

Supplementary Table S18.

Supplementary Table S19.

Supplementary Table S20.

## Data Availability

The supplementary files were available from Chao Luo (urologistluo2022@126.com).
