# Supplementary material for: Single-cell RNA sequencing reveals critical modulators of extracellular matrix of penile cavernous cells in erectile dysfunction
Source: Sci Rep. 2024 Mar 11;14:5886. doi: 10.1038/s41598-024-56428-0 (PMC10928087; doi:10.1038/s41598-024-56428-0)

Figure S1. The classification of cells in corpus cavernosum of penis. (A) Heatmap showed 20 main clusters. (B) UMAP showed the 20 main clusters. (C) Dotplot showed the cell type markers of 9 kinds of cells. (D) UMAP showed the cell distribution of 9 sample. (E) Each cell types exists in a different proportion of cells in nine samples.


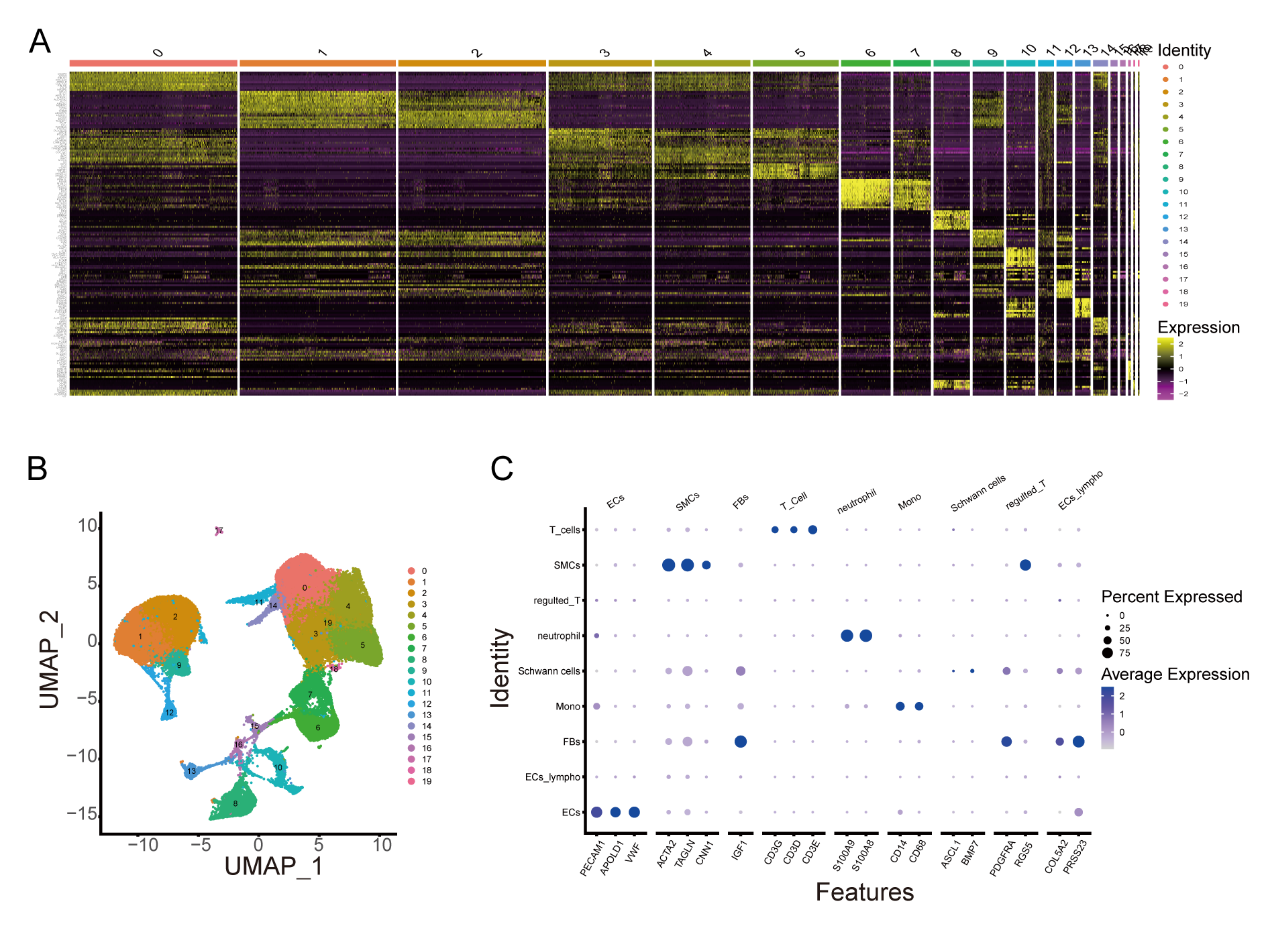


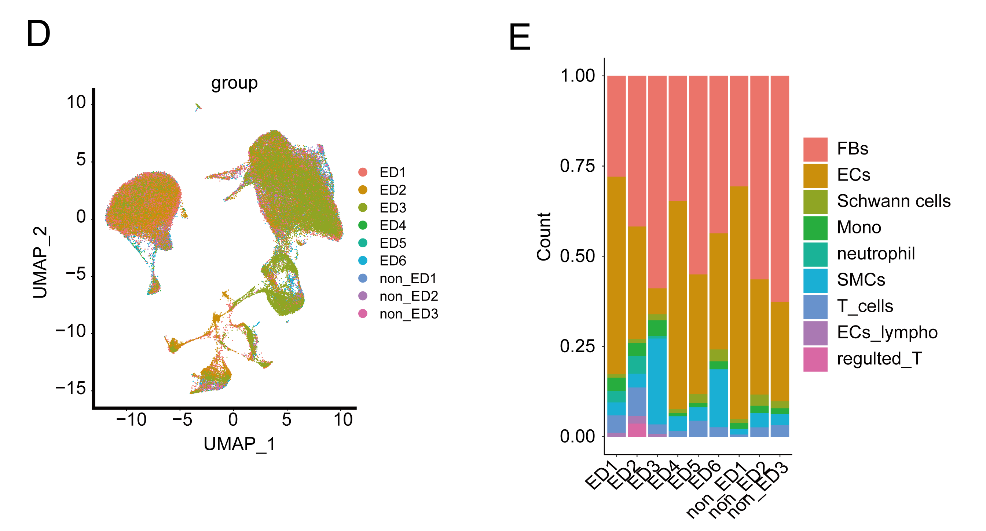


Figure S2. (**A**) UMAP showed the 11 subclusters of fibroblast based on the transcriptomically unique subpopulations. (**B**) Clustree plot evaluated clusterings at multiple resolutions. (**C**) Bar plot showed the relative proportions of subpopulations of fibroblast between the ED and non_ED samples. (**D**) Bar plot showed subpopulations f2 and f7 were enriched with up-regulated signature genes, whereas subpopulation f3, f5 and f6 was enriched in down-regulated signature genes. (**E**) GO enrichment analysis of upregulated genes.


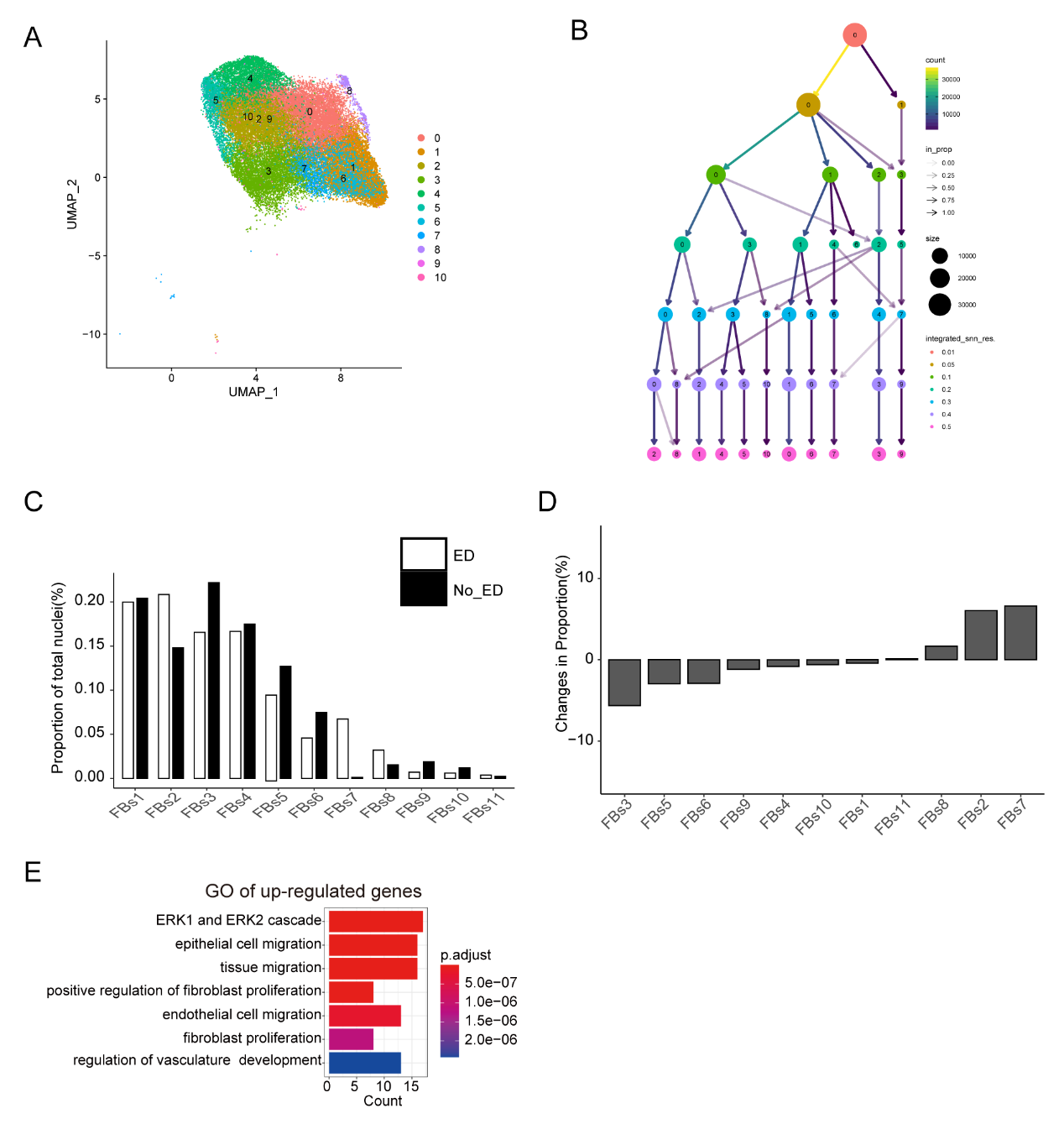


Figure S3. (**A**) UMAP showed the 7 subclusters of SMCs based on the transcriptomically unique subpopulations. (**B**) Clustree plot evaluated clusterings at multiple resolutions. (**C**) Bar plot showed the relative proportions of subpopulations of SMCs between the ED and non_ED samples. (**D**) Bar plot showed subpopulations s2 and s5 were enriched with up-regulated signature genes, whereas subpopulation s3 was enriched in down-regulated signature genes. (**E**) GO enrichment analysis of upregulated genes.


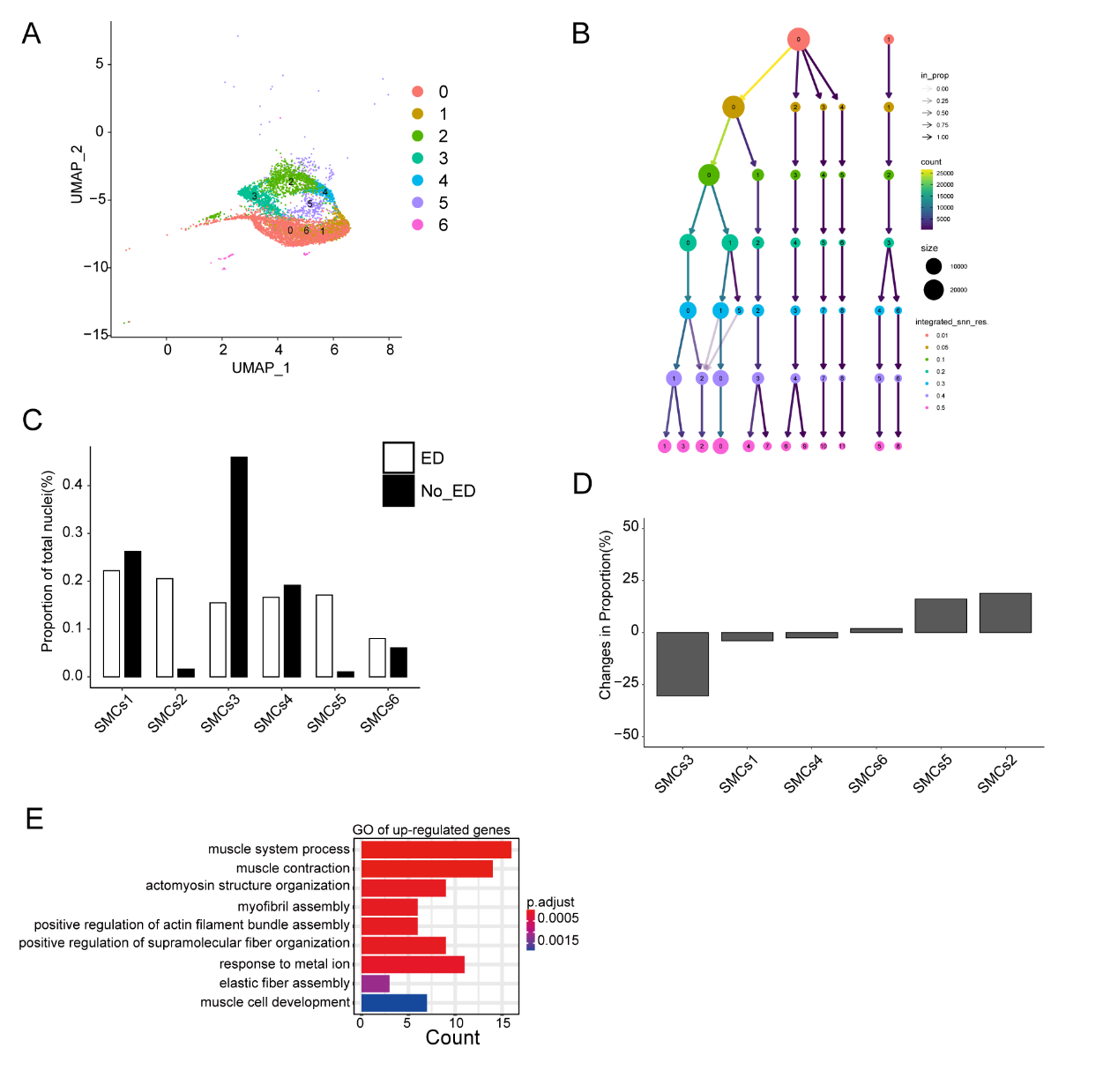


Figure S4. Figure S4 (**A**) UMAP showed the 9 subclusters of ECs based on the transcriptomically unique subpopulations. (**B**) Clustree plot evaluated clusterings at multiple resolutions. (**C**) Bar plot showed the relative proportions of subpopulations of ECs between the ED and non_ED samples. (**D**) Bar plot showed subpopulations e3 were enriched with up-regulated signature genes, whereas subpopulation e4 was enriched in down-regulated signature genes.


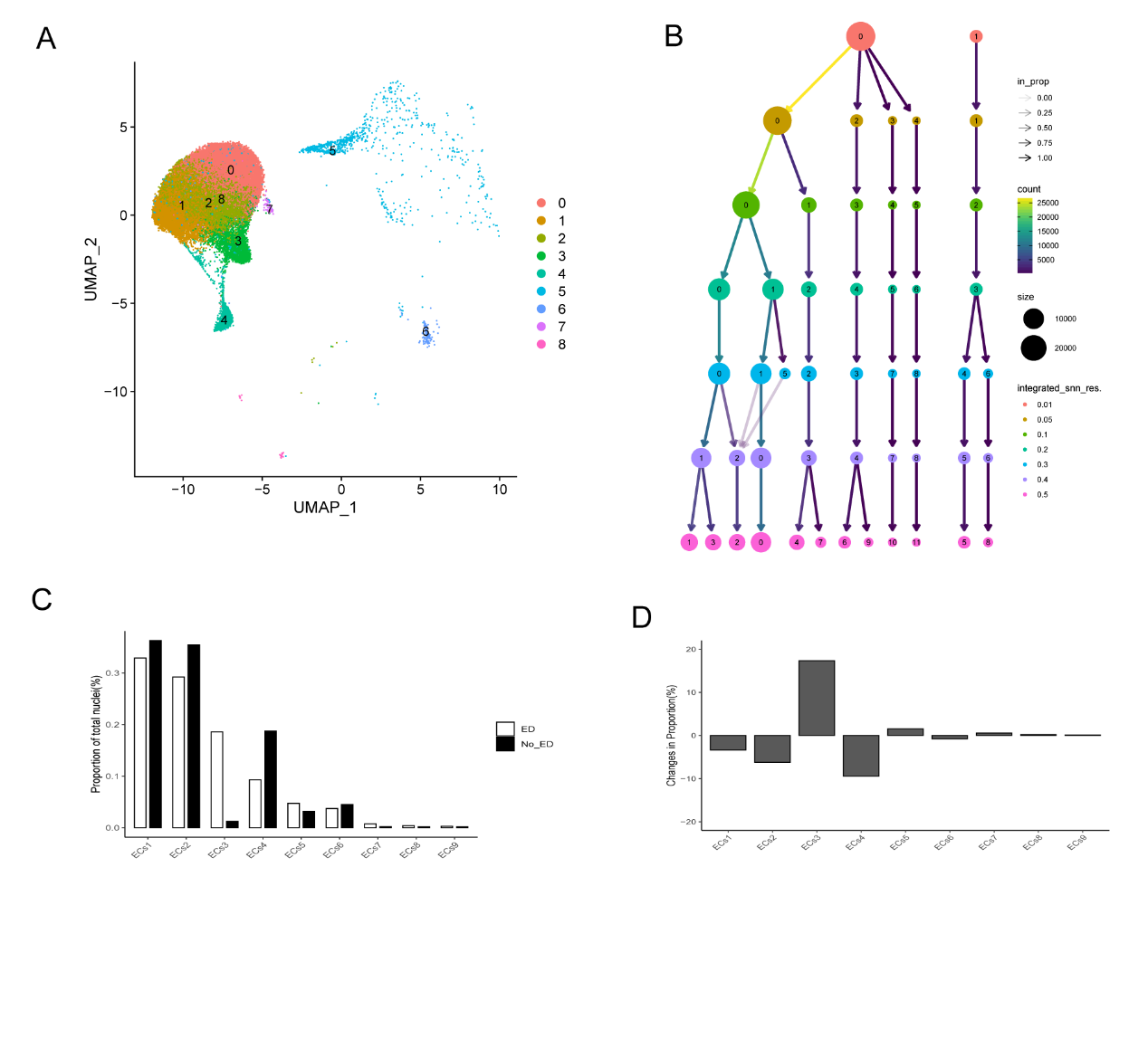


Fig.S5 Network plot of ligand-receptor pairs among each cell types of corpus cavernosum in ED (A) and non-ED (B), respectively.


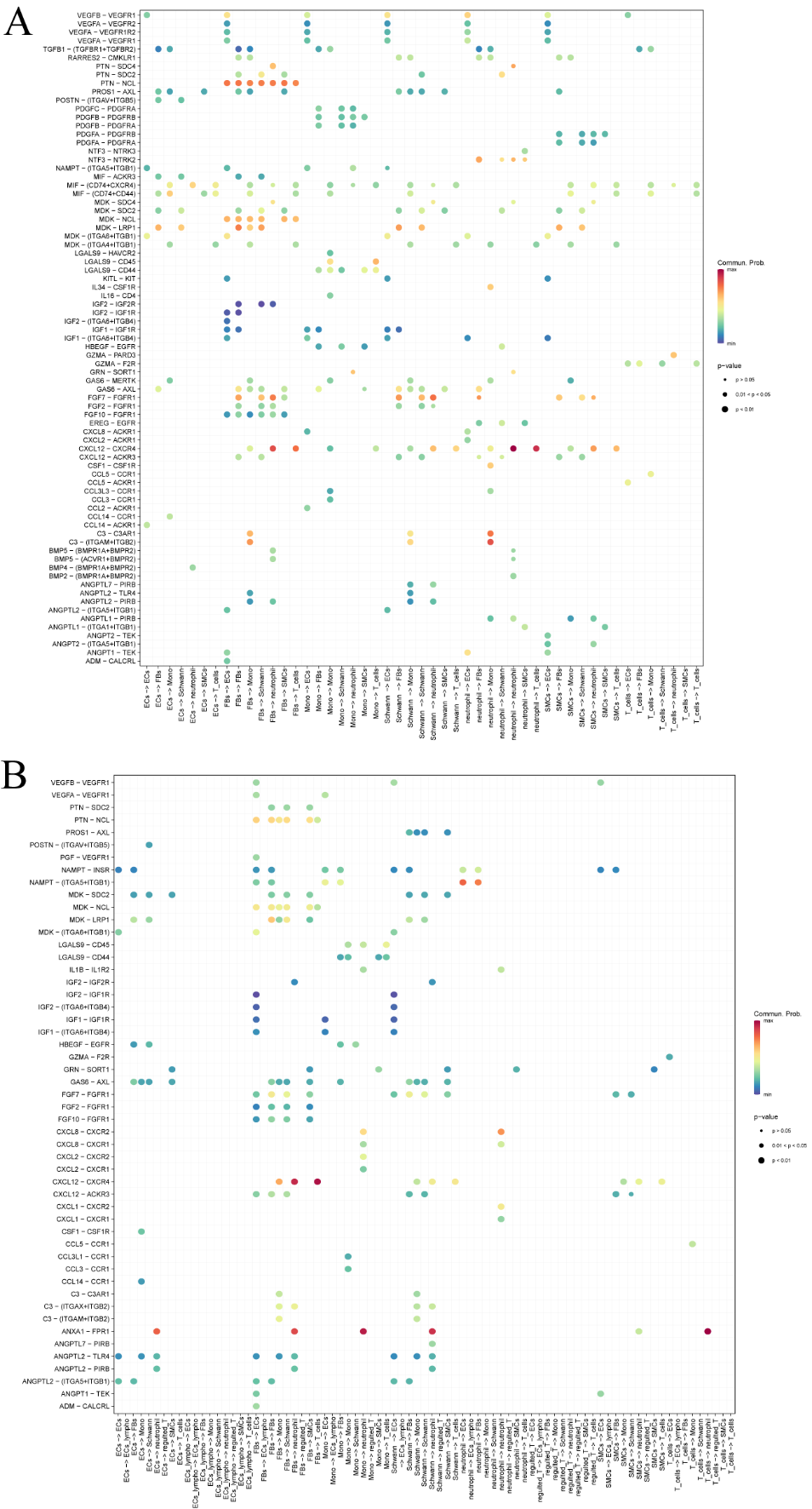

Supplement: Supplementary file 1 — Supplementary Figures. [file 41598_2024_56428_MOESM1_ESM.docx]
